# Exposure to patient violence towards mental health nurses: Emotional management, perception of the challenging patient, tolerance of violence, and the association with caring behaviors

**DOI:** 10.1192/j.eurpsy.2026.10954

**Published:** 2026-07-31

**Authors:** R. Shmilovitz, M. Itzhaki

**Affiliations:** Department of Nursing Sciences, School of Health Professions, Gray Faculty of Medical and Health Sciences, Tel Aviv University, Tel Aviv, Israel

## Abstract

**Introduction:**

Mental health nurses frequently encounter verbal and physical violence from patients while being required to demonstrate caring behaviors. These interactions generate complex emotional processes influenced by emotion management, tolerance of violence, and the patient’s perception as challenging.

**Objectives:**

This study examined caring behaviors among nurses in a mental health hospital, focusing on experienced and expected emotions, tolerance toward violence, and their perceptions of the patient as challenging. It further introduces a theoretical confrontation between Hochschild’s (1983) emotion management theory and Wolf’s (1994) caring theory.

**Methods:**

A mixed-methods design was employed with 60 registered mental health nurses. The quantitative phase included self-report questionnaires and a video vignette depicting verbal or physical patient violence. The qualitative phase comprised semi-structured interviews exploring nurses’ lived experiences of violence.

**Results:**

Reliability testing showed good to high internal consistency across study variables (α = .69–.94). Greater exposure to violence was associated with less expressive, physical, and deep emotion management. Positive perceptions of the patient as challenging and higher cognitive and deep emotion management were linked to more frequent caring behaviors. Regression analyses indicated that cognitive emotion management, positive patient perceptions, and prior violence-management training significantly predicted caring behaviors, explaining 43% of the variance. No significant differences were found between responses to verbal versus physical violence scenarios. A thematic analysis revealed that nurses working in psychiatric wards face multiple challenges stemming from patient violence directed towards them. The main areas of difficulty identified include: heavy workload, lack of specialized training to handle violent situations, and insufficient conditions to provide optimal care. Potential solutions to these challenges were identified as increasing staffing levels, improving hospitalization conditions, strengthening relationships between staff and patients, and developing strategies for preventing violence and responding effectively to challenging patients.

**Conclusions:**

The findings highlight the central role of emotion management and patient perception in sustaining caring behaviors, even under violent conditions. Cognitive and deep emotion management, along with a positive framing of patients as challenging, enhances caring practices. Violence-management training further strengthens caring responses. The study expands emotion management and caring theories into psychiatric nursing, underscoring the importance of training for preventing violence. Organizational support in fostering resilience can reduce burnout and ensure high-quality care.

**Disclosure of Interest:**

None Declared

